# Global epidemiology and antimicrobial resistance of extended-spectrum β-lactamase-producing Vibrio species: A One Health systematic review and meta-analysis

**DOI:** 10.14202/vetworld.2026.4210-4229

**Published:** 2026-09-22

**Authors:** Varangkana Thaotumpitak, Saran Anuntawirun, Nawaphorn Roongrojmongkhon, Manoj Tukaram Kamble, Saharuetai Jeamsripong

**Affiliations:** 1 Department of Microbiology, Faculty of Public Health, Mahidol University, Bangkok 10400, Thailand.; 2 Department of Veterinary Public Health, Faculty of Veterinary Science, Chulalongkorn University, Bangkok 10330, Thailand.; 3 Department of Agricultural and Fishery Science, Faculty of Science and Technology, Prince of Songkla University, Pattani 94000, Thailand.; 4 Research Unit in Microbial Food Safety and Antimicrobial Resistance, Department of Veterinary Public Health, Faculty of Veterinary Science, Chulalongkorn University, Bangkok 10330, Thailand.

**Keywords:** antimicrobial resistance, aquatic environment, extended-spectrum β-lactamase, meta-analysis, multidrug resistance, One Health, systematic review, Vibrio species

## Abstract

**Background and Aim:**

*Vibrio* spp. are important aquatic bacteria associated with human disease, aquaculture losses, seafood safety, and environmental reservoirs of antimicrobial resistance (AMR). The emergence of extended-spectrum β-lactamase (ESBL)-producing *Vibrio* spp. may compromise β-lactam therapy and facilitate resistance dissemination across interconnected One Health sectors. This systematic review and meta-analysis aimed to determine the prevalence of *Vibrio* spp. and ESBL-producing isolates and characterize their phenotypic AMR, multidrug resistance (MDR), and resistance-associated genes across human, animal, and environmental sources.

**Materials and Methods:**

Literature published from 2012 to 2024 was systematically retrieved from PubMed, Scopus, and Google Scholar following Preferred Reporting Items for Systematic Reviews and Meta-Analyses guidelines. The protocol was registered in the International Prospective Register of Systematic Reviews (CRD420251114318). Pooled prevalence estimates were calculated using random-effects models, heterogeneity was assessed using Cochran’s Q and I² statistics, and subgroup analyses were conducted by continent and *Vibrio* species. Risk-of-bias was evaluated using the Joanna Briggs Institute checklist.

**Results:**

Of 1,120 records identified, 31 studies were included, representing 4,486 samples and 3,242 *Vibrio* isolates, predominantly from Asia and Africa. The pooled prevalence of *Vibrio* spp. was 48.3% (95% confidence interval [CI]: 34.0–62.7%; I² = 99.37%), whereas ESBL-producing *Vibrio* spp. had a pooled prevalence of 19.4% (95% CI: 3.2–43.7%; I² = 95.87%). Ampicillin showed the highest pooled β-lactam resistance at 73.6% (95% CI: 58.4–86.4%). The overall pooled MDR prevalence was 31.5% (95% CI: 6.1–64.7%). Resistance varied substantially among *Vibrio* species, including marked resistance to third-generation cephalosporins in selected species. Among resistance determinants, *bla*_TEM_ was predominant, with a pooled prevalence of 33.8%, while carbapenemase-associated *bla*_VIM_ and *bla*_IMP_ were also detected. Considerable between-study heterogeneity was observed for most outcomes.

**Conclusion:**

ESBL-producing and multidrug-resistant *Vibrio* spp. occur across human, animal, and environmental sources, demonstrating substantial geographical and species-specific variation. The high β-lactam resistance and detection of clinically important resistance determinants emphasize the need for harmonized antimicrobial susceptibility testing, molecular surveillance, and integrated One Health monitoring to limit the emergence and dissemination of resistant *Vibrio* spp.

## INTRODUCTION

Antimicrobial resistance (AMR) has emerged as one of the most pressing global health challenges, undermining antimicrobial therapy and threatening control of infectious diseases across human, animal, and environmental domains [1, 2]. Within this context, *Vibrio* spp. are Gram-negative bacteria of substantial public health importance. Toxigenic *Vibrio cholerae* remains a major cause of cholera worldwide, with more than 100,000 cholera-related deaths estimated in 2019, while AMR has increasingly been reported in other clinically important species, including *Vibrio parahaemolyticus*, for which resistance to glycopeptides and β-lactam antibiotics has exceeded 50% [3, 4]. Among Gram-negative bacteria, resistance to β-lactam antibiotics is particularly concerning because of the global dissemination of extended-spectrum β-lactamases (ESBLs) [1, 5]. These enzymes hydrolyze a broad spectrum of β-lactam antibiotics, including third- and fourth-generation cephalosporins, thereby restricting therapeutic options and potentially contributing to treatment failure and an increased healthcare burden [1, 6].

*Vibrio* species are ubiquitous aquatic bacteria inhabiting marine, estuarine, and freshwater environments [7–10]. Several species are important pathogens of humans and animals, causing clinical manifestations ranging from mild gastroenteritis to life-threatening septicemia [11–13]. Humans commonly become infected by consuming contaminated seafood, particularly raw or undercooked aquatic products, and through direct contact with contaminated water or infected aquatic animals [14, 15]. In aquaculture systems, *Vibrio* spp. can also cause economically important diseases in fish and shellfish, resulting in production losses and increased antimicrobial use [16]. Their close association with aquatic environments, aquaculture systems, seafood production, and environmental exposure pathways places *Vibrio* spp. at the interface of human, animal, and environmental health [17, 18]. Aquatic environments can facilitate the persistence and exchange of AMR determinants among bacterial populations, particularly in areas affected by aquaculture activities, wastewater discharge, and other anthropogenic pressures. Consequently, antimicrobial-resistant *Vibrio* spp. in these interconnected settings matter not only for clinical treatment but also for food safety and the environmental spread of resistance.

ESBL-producing *Vibrio* spp. have increasingly been reported from diverse sources, including clinical specimens, aquatic animals, seafood products, and environmental samples [19, 20]. ESBL-associated genes, including *bla*_CTX-M_, *bla*_TEM_, and *bla*_SHV_ have been identified in several *Vibrio* species, demonstrating their capacity to acquire and maintain clinically important resistance determinants [21]. In addition to ESBL production, multidrug resistance (MDR), generally defined as acquired non-susceptibility to at least one agent in three or more antimicrobial categories, has been reported among *Vibrio* spp., further restricting therapeutic options and complicating infection management [3, 14]. Mobile genetic elements, including plasmids, integrons, and transposons, may mediate the dissemination of resistance determinants and facilitate horizontal gene transfer within aquatic microbial communities [22, 23]. Furthermore, aquatic environments affected by antimicrobial use, aquaculture activities, and wastewater contamination may serve as reservoirs for ESBL-producing and other antimicrobial-resistant *Vibrio* spp., creating potential pathways for the persistence and transmission of resistance across One Health sectors [23, 24].

Despite the increasing number of reports describing AMR in *Vibrio* spp., important gaps remain in understanding the global epidemiology and One Health distribution of ESBL-producing *Vibrio* spp. Available evidence is geographically heterogeneous and fragmented across clinical, animal, food, and environmental settings, with individual studies commonly focusing on a particular *Vibrio* species, sample source, antimicrobial class, or resistance determinant [21]. Consequently, the extent to which ESBL-producing and multidrug-resistant *Vibrio* spp. are distributed across interconnected human, animal, and environmental reservoirs remains unclear. The previous systematic review was based on a single bibliographic database and no longer captures the expanding body of evidence [21]. More importantly, no previous meta-analysis has simultaneously quantified the prevalence of ESBL-producing *Vibrio* spp., broader phenotypic AMR patterns, MDR, and ESBL-associated resistance genes across the three One Health sectors. Species-specific and geographical variations in resistance also remain insufficiently characterized, limiting comparisons among epidemiological settings and the identification of potential resistance hotspots. An updated quantitative synthesis integrating epidemiological, phenotypic, and genotypic evidence is therefore needed to define the magnitude and distribution of ESBL-producing and other antimicrobial-resistant *Vibrio* spp. and to clarify their potential public health significance within a One Health framework.

This systematic review and meta-analysis aimed to synthesize available evidence on the prevalence of *Vibrio* spp. and ESBL-producing *Vibrio* spp. and to characterize their phenotypic AMR profiles, MDR, and resistance-associated genes across human, animal, and environmental sources. Specifically, the study sought to quantify the pooled prevalence of *Vibrio* spp. and ESBL-producing isolates; determine pooled resistance estimates for clinically relevant antimicrobial agents and MDR; characterize the distribution of ESBL- and other resistance-associated genes; and evaluate geographical and species-specific variations in resistance. By integrating evidence across interconnected One Health sectors, this study aimed to provide a comprehensive assessment of the epidemio-logical distribution and resistance characteristics of *Vibrio* spp. and to generate evidence that can support risk assessment, harmonized AMR surveillance, and future strategies for monitoring and mitigating the dissemination of antimicrobial-resistant *Vibrio* spp.

## MATERIALS AND METHODS

### Ethical approval

This systematic review and meta-analysis was reviewed by the Human Research Ethics Committee, Faculty of Public Health, Mahidol University (Protocol No. MUPH 45/2026), which determined that the study protocol complies with the criteria for the “research with exemption category”. As the study synthesized and analyzed data from previously published studies and did not involve direct recruitment of human participants, collection of identifiable personal information, or direct experimentation on animals, additional consent was not required.

### Study period and literature sources

Data extraction and statistical analyses were conducted from April to May 2026. The review included eligible studies published from January 1, 2012, to December 31, 2024. The electronic literature search was conducted using PubMed, Scopus, and Google Scholar to identify eligible articles published within the predefined study period. In addition, the reference lists of all included studies were manually screened to identify potentially eligible publications that were not retrieved through the electronic database searches.

### Study design and reporting standards

This study was conducted as a systematic review and meta-analysis in accordance with the Preferred Reporting Items for Systematic Reviews and Meta-Analyses (PRISMA) 2020 guidelines [25]. The review protocol was prospectively registered in the International Prospective Register of Systematic Reviews (PROSPERO; registration No. CRD420251114318). During the review process, the PROSPERO record was amended to include the additional keywords without any substantive modification of the registered methodological protocol.

### Literature search

The literature search strategy was developed using a modified Population, Exposure, Comparator, and Outcome (PECO) framework [26]. The strategy was designed to identify both phenotypic and genotypic evidence of extended-spectrum β-lactamase (ESBL)-producing *Vibrio* spp. across human, animal/aquaculture, food, and environmental sources. Controlled vocabulary and free-text terms related to *Vibrio* spp. and ESBL production were combined to maximize search sensitivity.

The core Boolean search strategy was structured as follows: “*Vibrio*” AND (“ESBL” OR “extended-spectrum β-lactamases” OR “extended-spectrum beta lactamase” OR “*bla*_CTX-M_” OR “*bla*_TEM_” OR “*bla*_SHV_”). Additional species-specific terms, including *Vibrio parahaemolyticus*, *Vibrio vulnificus*, and *Vibrio cholerae*, were incorporated where appropriate, with the search syntax and fields adapted to the requirements of each database. Searches were restricted to original research articles published in English during the predefined publication period. Complete database-specific search strategies, including Boolean operators, search fields, and applied filters, are provided in Table S1.

### Definition and classification of ESBL-producing *Vibrio* spp.

A *Vibrio* isolate was classified as ESBL-producing when phenotypic ESBL production was confirmed and/or an ESBL-associated gene was detected using a molecular method, regardless of whether phenotypic testing was performed. Phenotypic confirmation included standard ESBL confirmatory methods reported in the original studies, such as the double-disk synergy test, combined-disk test using third-generation cephalosporins with and without clavulanic acid, ESBL E-test, or minimum inhibitory concentration (MIC)-based confirmation demon-strating reduced cephalosporin susceptibility with clavulanate inhibition. Where specified by the original studies, interpretation was based on recognized criteria, including those of the Clinical and Laboratory Standards Institute (CLSI) or European Committee on Antimicrobial Susceptibility Testing (EUCAST). Molecular confirmation included the detection of ESBL-associated β-lactamase genes, including *bla*_CTX-M_, *bla*_TEM_, *bla*_SHV_, and other ESBL-associated genes reported by the included studies.

To evaluate the potential influence of this broader ESBL-definition on the pooled prevalence estimate, a sensitivity analysis was performed by restricting the analysis to studies reporting phenotypically confirmed ESBL-producing isolates.

### Eligibility criteria

Studies were eligible if they investigated *Vibrio* isolates obtained from clinical cases or healthy humans; animals, including aquaculture species and terrestrial livestock; food or seafood products; or environmental sources, including water and sediment. Studies were required to report phenotypic confirmation of ESBL production using recognized methods and/or molecular detection of ESBL-associated genes or their variants. No geographical restrictions were applied, but only original research articles published in English were eligible.

Review articles, meta-analyses, editorials, conference abstracts, short communications without sufficient primary data, and case reports lacking data suitable for prevalence estimation were excluded.

### Outcomes

The primary outcome was the prevalence of ESBL-producing *Vibrio* spp. Secondary outcomes included the prevalence and distribution of *Vibrio* spp., distribution of ESBL-associated genes and their variants, phenotypic AMR profiles, and MDR, where reported. Geographic, source-specific, and species-specific patterns were also evaluated where sufficient comparable data were available.

### Study selection and data extraction

All retrieved records were imported into reference-management software, and duplicate records were removed. Titles and abstracts were independently screened by two reviewers (VT and SA), followed by full-text assessment of potentially eligible articles. Disagreements were resolved through discussion and consensus, with consultation with a third reviewer (SJ) when necessary. Reasons for exclusion at the full-text stage were documented, and the overall study selection process was summarized using a PRISMA flow diagram.

Data were independently extracted by two reviewers using a standardized and piloted extraction form. The variables collected included publication details, geographical location and continent, study design, sampling strategy, sample source, *Vibrio* species, species identification methods, total number of samples tested, total number of *Vibrio* isolates tested, number of ESBL-positive isolates, phenotypic and molecular ESBL-detection methods, ESBL-associated genes and variants, antimicrobial susceptibility testing (AST) methods, and interpretative standards, including CLSI and EUCAST criteria where reported. A detailed list of the extracted variables is provided in Supplementary File S2. When relevant information was missing or unclear, attempts were made to contact the corresponding authors for clarification.

### Risk-of-bias and quality assessment

The methodological quality and risk-of-bias of the included studies were independently assessed by VT and SA using the Joanna Briggs Institute Critical Appraisal Checklist for Studies Reporting Prevalence Data [27]. The checklist assesses the appropriateness of the sampling frame and sampling method, adequacy of sample size, description of study participants and setting, coverage of the identified sample in the analysis, validity of methods used to identify the condition, reliability of measurement, appropriateness of statistical analysis, and adequacy of the response rate or management of incomplete data. Each domain was rated as “yes,” “no,” “unclear,” or “not applicable.”

For descriptive classification, the overall risk-of-bias was categorized according to the number of applicable domains rated “yes”: low risk (7–9 points), moderate risk (4–6 points), and high risk (0–3 points). Risk-of-bias findings were incorporated into sensitivity analyses. Where a meta-analysis included studies classified as having a high risk of bias, the analysis was repeated after excluding these studies. The resulting pooled prevalence estimates, 95% confidence intervals (CIs), and heterogeneity statistics were compared with those from the primary analyses to assess the robustness of the findings. Additional sensitivity analyses excluding studies with small sample sizes were performed where applicable.

### Data synthesis and meta-analysis

A narrative synthesis was initially performed to summarize study characteristics, geographical distribution, *Vibrio* species, ESBL-associated gene profiles, and AMR patterns. Where sufficiently comparable quantitative data were available, meta-analyses were conducted to generate pooled prevalence estimates.

The denominator was defined according to the outcome being analyzed. For the pooled prevalence of *Vibrio* spp., the numerator was the number of *Vibrio*-positive samples and the denominator was the total number of samples tested. ESBL production and AMR were analyzed as isolate-based outcomes. For ESBL prevalence, the numerator was the number of ESBL-positive *Vibrio* isolates and the denominator was the total number of *Vibrio* isolates tested for ESBL production. For individual antimicrobial agents, the numerator was the number of resistant *Vibrio* isolates and the denominator was the number of *Vibrio* isolates tested against the corresponding antimicrobial agent.

Prevalence proportions were logit-transformed to stabilize variances and pooled using a random-effects model with restricted maximum likelihood (REML) estimation of between-study variance. No continuity correction was applied to studies reporting zero events. Pooled estimates are presented with 95% CIs.

### Assessment of heterogeneity and subgroup analyses

Statistical heterogeneity among studies was assessed using Cochran’s Q test and quantified using the I² statistic. I² values of approximately 25%, 50%, and 75% were interpreted as indicating low, moderate, and high heterogeneity, respectively.

Pre-specified subgroup analyses were conducted to investigate potential sources of heterogeneity. Subgroup meta-analyses by continent were performed for the pooled prevalence of *Vibrio* spp. and ESBL-producing *Vibrio* spp., and additional analyses were stratified by *Vibrio* species. To minimize unstable estimates, subgroup meta-analysis was undertaken only when at least two studies were available within a subgroup and the numerator and denominator were clearly reported. Sensitivity analyses were also conducted by sequentially excluding studies with high prevalence estimates to evaluate the robustness of the pooled results.

### Publication bias

Potential publication bias was evaluated separately for each meta-analyzed outcome. Funnel plots, Galbraith plots, and Egger’s regression test were used when an outcome included at least three studies. All statistical analyses were performed using Stata version 19 (StataCorp LLC, College Station, TX, USA). A two-sided p < 0.05 was considered statistically significant.

## RESULTS

### Search results

A total of 1,120 records were identified through PubMed (n = 44), Scopus (n = 80), and Google Scholar (n = 996). After removal of 40 duplicate records, 1,080 records remained for title and abstract screening. Of these, 847 were excluded because they were irrelevant (n = 810) or published in languages other than English (n = 37). Thirteen records could not be retrieved, leaving 220 full-text articles for eligibility assessment.

Of the 220 full-text articles assessed, 189 were excluded for the following reasons: outcomes were irrelevant (n = 48), studies were unrelated to ESBL or *Vibrio* spp. (n = 99), theses (n = 15), reviews or notes (n = 9), letters or conference papers (n = 9), case reports (n = 5), book chapters (n = 1), short communications (n = 1), and studies involving only one isolate (n = 2). Finally, 31 studies were included in the systematic review and meta-analysis (Figure 1).

**Figure 1:**
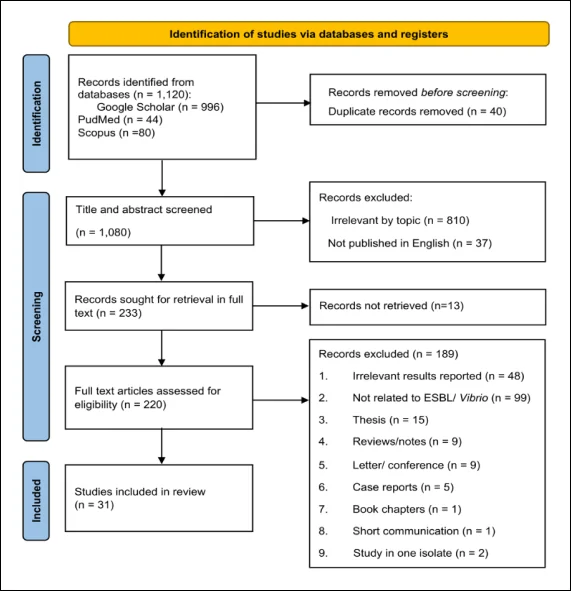
PRISMA flow diagram of the study selection process for *Vibrio* and AMR studies. PRISMA = Preferred Reporting Items for Systematic Reviews and Meta-Analyses; AMR = Antimicrobial resistance.

### Study characteristics

The 31 included studies were published between 2012 and 2024. The most were published in 2022 (n = 8), followed by 2023 (n = 4) and 2024 (n = 4). Geographically, studies were concentrated in Asia (n = 19) and Africa (n = 8), with fewer studies conducted in Europe (n = 2), North America (n = 1), and South America (n = 2). Overall, the included studies represented 19 countries. Thailand (n = 5), China (n = 4), and India (n = 3) contributed the most studies, while most other countries were represented by a single study.

Only studies reporting both numerator and denominator data were included in the respective prevalence meta-analyses. Specifically, 11 studies comprising 4,486 samples reported the exact numbers of *Vibrio*-positive samples and total samples examined and were included in the pooled prevalence analysis of *Vibrio* spp. (Table 1) [8, 28–57]. Studies contributing to the pooled prevalence analysis of ESBL-producing *Vibrio* spp. similarly reported the exact number of ESBL-positive *Vibrio* isolates and the corresponding number of *Vibrio* isolates tested for ESBL production (Table S2). Among studies with quantitative prevalence data, the number of samples examined varied considerably, ranging from 16 to 2,051.

**Table 1:** Characteristics of studies included in the systematic review and meta-analysis (n = 31)

**Author**	**Year**	**Study duration**	**Country**	**Continent**	**Sample source**	**Positive samples (n)**	**Total samples (n)**
Afum *et al.* [28]	2022	2017–2021	Ghana	Africa	Clinical human	28	792
Amin *et al.* [29]	2024	2020–2021	Bangladesh	Asia	Aquatic animals	312	500
Baah *et al.* [30]	2022	NA	Ghana	Africa	Livestock	10	40
Baron *et al.* [31]	2017	2000–2001	France	Europe	Environment	NA	NA
Beshiru *et al.* [32]	2018	2016–2017	Nigeria	Africa	Aquatic animals	NA	1,440*
Canellas *et al.* [33]	2021	2018–2019	Brazil	South America	Environment	NA	66*
Changsen *et al.* [34]	2023	NA	Thailand	Asia	Aquatic animals	NA	190*
Chiyangi *et al.* [35]	2017	NA	Zambia	Africa	Clinical human	111	271
Dahanayake *et al.* [36]	2019	NA	Korea	Asia	Aquatic animals	NA	NA
Gupta *et al.* [37]	2022	2002–2016	India	Asia	Clinical human	NA	NA
Gxalo *et al.* [8]	2021	2018	South Africa	Africa	Environment	NA	72*
Huynh *et al.* [38]	2019	NA	Vietnam	Asia	Aquatic animals	80	120
Jeamsripong *et al.* [39]	2022	2021–2022	Thailand	Asia	Environment	NA	NA
Jubyda *et al.* [40]	2023	2015–2021	Bangladesh	Asia	Clinical human	NA	NA
Li *et al.* [41]	2015	2013	China	Asia	Livestock	NA	318*
Lian *et al.* [42]	2024	2020	China	Asia	Mixed	NA	NA
Lajnef *et al.* [43]	2024	NA	Tunisia	Africa	Aquatic animals	NA	NA
Mogessie *et al.* [44]	2024	2023	Ethiopia	Africa	Environment	39	206
Narayanan *et al.* [45]	2020	NA	India	Asia	Mixed	90	100
Onohuean *et al.* [46]	2022	2019	Uganda	Africa	Environment	NA	NA
Pazhani *et al.* [47]	2014	NA	India	Asia	Clinical human	NA	13,607*
Raharjo *et al.* [48]	2023	NA	Thailand	Asia	Aquatic animals	NA	93*
Saifedden *et al.* [49]	2016	NA	Malaysia	Asia	Aquatic animals	NA	NA
Shah *et al.* [50]	2012	2010–2011	Nepal	Asia	Clinical human	22	238
Tate *et al.* [51]	2022	2019	USA	North America	Aquatic animals	NA	1,634*
Thaotumpitak *et al.* [52]	2023	2019–2020	Thailand	Asia	Aquatic animals	NA	NA
Yen *et al.* [53]	2020	2018	Vietnam	Asia	Aquatic animals	80	120
Yano *et al.* [54]	2014	2007–2009	Thailand	Asia	Aquatic animals	23	48
Yu *et al.* [55]	2022	2002–2020	China	Asia	Aquatic animals	NA	NA
Valdez *et al.* [56]	2022	2020	Portugal	Europe	Aquatic animals	NA	NA
Zheng *et al.* [57]	2021	2015–2017	China	Asia	Mixed	593	2,051
Total						1,388	4,486

NA = Not available. *Studies excluded from the pooled prevalence analysis of *Vibrio*-positive samples because numerator data were unavailable. The totals of 1,388 positive samples and 4,486 total samples refer to studies providing the numerator and denominator data required for the pooled prevalence analysis and do not represent the sum of all numerical values reported across the 31 included studies.

Among the 4,486 samples contributing to the pooled prevalence analysis, sample sources were grouped into three One Health sectors: animal-derived samples (n = 2,899), human samples (n = 1,301), and environmental samples (n = 286). Animal-derived samples included aquatic animals, seafood, meat, and livestock products, whereas environmental samples primarily comprised water samples. Mixed-source studies were classified according to the source-specific sample numbers reported by the original investigators.

Most studies isolated *Vibrio* spp. using procedures consistent with the U.S. Food and Drug Administration Bacteriological Analytical Manual, typically involving enrichment in alkaline peptone water followed by selective culture on thiosulfate-citrate-bile salts-sucrose (TCBS) agar. Alternative selective media included chromogenic agar, marine agar, and thiosulfate-taurocholate-gelatin agar (TTGA).

Species identification was performed using one or more confirmatory methods. Conventional biochemical testing, either alone or in combination with commercial identification systems such as Analytical Profile Index, was the most frequently reported approach (n = 15). Polymerase chain reaction (PCR)-based molecular confirmation targeting species-specific genes, including *toxR*, *tl*, and *hsp60*, was reported in nine studies. Matrix-assisted laser desorption/ionization time-of-flight (MALDI-TOF) mass spectrometry was used in six studies, whereas VITEK-based automated identification systems were used in two studies. Two studies did not clearly report the species-confirmation method.

For AST, the Kirby–Bauer disk diffusion method was the most frequently used approach (n = 22), whereas agar dilution and broth microdilution were each reported in four studies. Multiple editions of CLSI guidelines were used, with the 2015 edition cited most frequently (n = 5). Two studies applied interpretative criteria based on EUCAST guidelines. The most frequently reported quality-control strain was *Escherichia coli* American Type Culture Collection (ATCC) 25922. Additional control strains included *Staphylococcus aureus* ATCC 29213, *Pseudomonas aeruginosa* ATCC 27853, and *Klebsiella pneumoniae* ATCC 700603. However, 17 studies did not report the quality-control strains used.

The *Vibrio* species identified among the included studies were diverse (Table S2). The most frequently reported species was *V. parahaemolyticus* (n = 10 studies), followed by *V. cholerae* (n = 9), unspecified *Vibrio* spp. (n = 5), *V. vulnificus* (n = 3), and *Vibrio alginolyticus* (n = 3). Less frequently reported species included *Vibrio diabolicus*, *Vibrio mimicus*, *Vibrio fluvialis*, *Vibrio navarrensis*, and *Vibrio scophthalmi*.

### Risk-of-bias assessment

Of the 31 included studies, 15 were classified as having a low risk-of-bias and 16 as having a moderate risk-of-bias; none were classified as having a high overall risk-of-bias (Table S3). At the domain-level, favorable ratings were observed for an appropriate sampling frame (Q1; 83.9%), adequate description of study participants and settings (Q4; 67.7%), adequate coverage of the identified sample in the analysis (Q5; 100%), valid methods for identification of the condition (Q6; 96.8%), reliable outcome measurement (Q7; 93.5%), and appropriate statistical analysis (Q8; 100%) (Figure 2).

In contrast, high risk ratings were more frequent for sampling method (Q2; 58.1%) and sample size adequacy (Q3; 45.2%), while 12.9% of studies had an unclear risk-of-bias for sample size adequacy. The response-rate domain (Q9) was considered not applicable to all included studies. Moderate overall risk-of-bias classifications were therefore primarily associated with limitations in sampling strategy, sample size adequacy, and representativeness.

**Figure 2:**
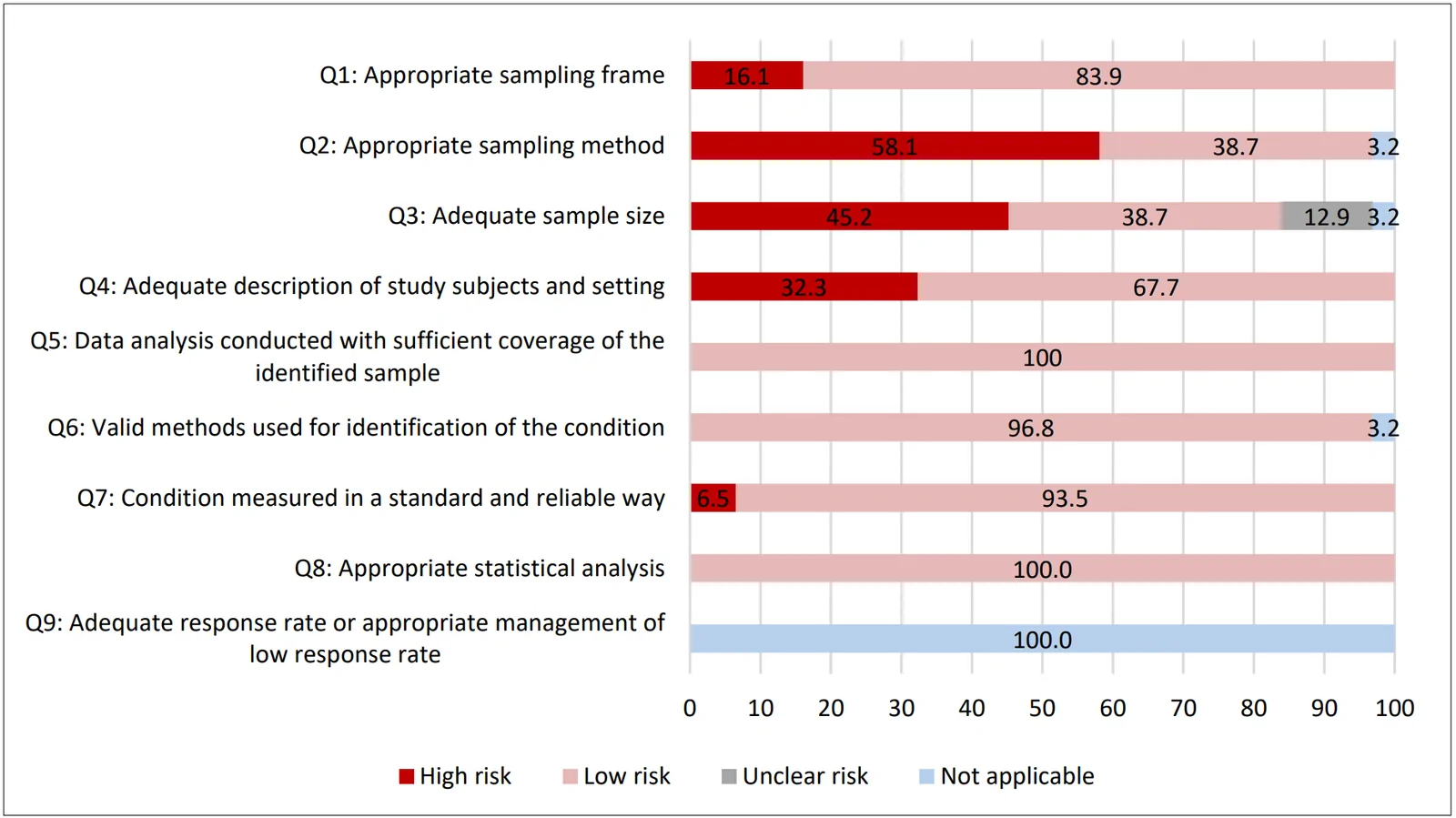
Domain-level risk-of-bias assessment of included studies using the JBI Critical Appraisal Checklist. JBI = Joanna Briggs Institute.

### Pooled prevalence of *Vibrio* spp. and heterogeneity

The pooled prevalence of *Vibrio* spp. was estimated from 11 studies, comprising 4,486 samples. The overall pooled prevalence was 48.3% (95% CI: 34.0–62.7%), with substantial between-study heterogeneity (I² = 99.37%, p < 0.001; Figure 3A).

Geographically, the pooled prevalence was higher in Asia at 56.7% (95% CI: 40.9–72.5%) than in Africa at 21.8% (95% CI: 6.1–37.6%). Considerable heterogeneity persisted within both continental subgroups, with I² values of 98.72% for Asia and 97.58% for Africa. The test for subgroup differences was statistically significant (Qb = 9.39, p < 0.001), indicating significant variation in pooled prevalence between continents.

**Figure 3:**
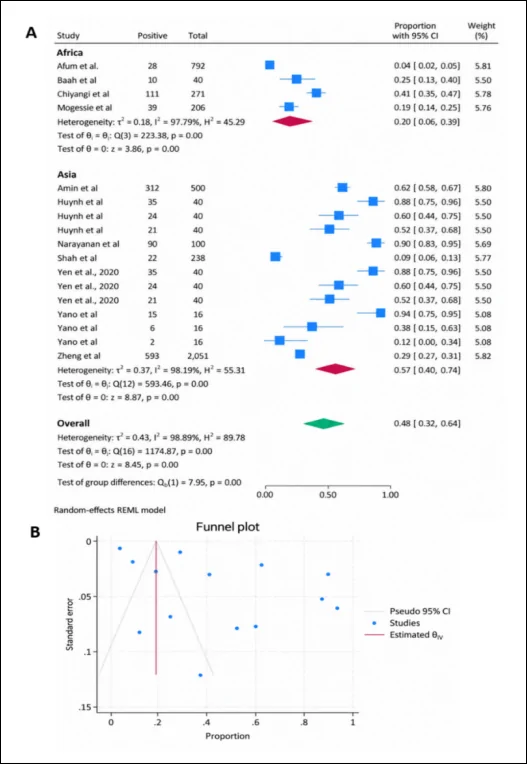
Meta-analysis of the pooled prevalence of *Vibrio* spp. and assessment of heterogeneity. (A) Forest plot showing pooled prevalence estimates generated using a random-effects REML model and stratified by continent. Squares represent individual study estimates, with sizes proportional to study weight; horizontal lines represent 95% confidence intervals; and diamonds represent pooled estimates. (B) Funnel plot showing the relationship between prevalence estimates and their standard errors. The central vertical line represents the pooled effect estimate, and the triangular region represents the pseudo 95% confidence limits. REML = Restricted maximum likelihood.

The funnel plot showed an asymmetric distribution of study estimates around the pooled effect estimate, suggesting possible publication bias or small-study effects (Figure 3B). Several studies with relatively large standard errors were distributed away from the central estimate, and some estimates fell outside the pseudo 95% confidence limits. Thus, smaller studies appeared to show greater variability in prevalence estimates.

Leave-one-out sensitivity analysis showed that exclusion of Yano *et al.* [54] produced no meaningful change in pooled prevalence, which remained at 48.3% (Supplementary File S4). The τ² value decreased slightly from 0.087 to 0.082, while I² remained essentially unchanged (99.37% vs. 99.4%), indicating that this study did not substantially influence the overall pooled estimate.

### Pooled prevalence of ESBL-producing *Vibrio* spp. and heterogeneity

Based on five studies comprising 388 isolates, the pooled prevalence of ESBL-producing *Vibrio* spp. was 19.4% (95% CI: 3.2–43.7%) using a random-effects model (Figure 4A). Study-specific estimates ranged from 0.0% to 74.1%, with the highest prevalence reported by Narayanan *et al.* [45], whereas two datasets reported no ESBL-producing *Vibrio* isolates. Because of the small number of contributing studies and their geographical concentration, including three studies from Asia, continent-specific subgroup analysis was not performed.

**Figure 4:**
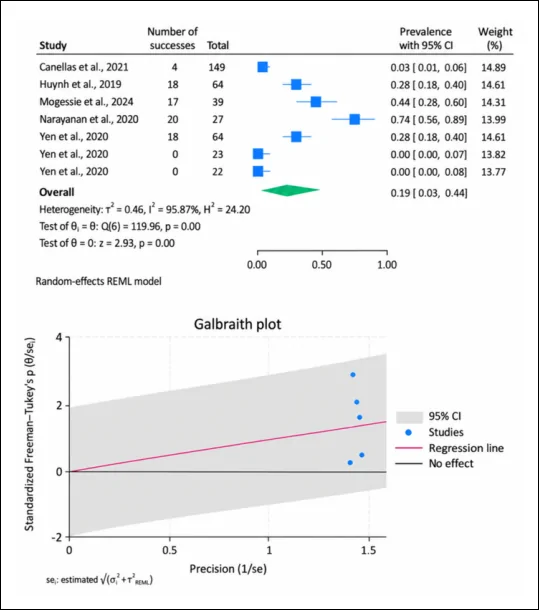
Meta-analysis of the pooled prevalence of ESBL-producing *Vibrio* spp. and heterogeneity assessment. (A) Forest plot showing pooled prevalence generated using a random-effects REML model. Squares represent individual study estimates, with sizes proportional to study weight; horizontal lines indicate 95% confidence intervals; and the diamond represents the pooled estimate. (B) Galbraith plot showing the relationship between standardized effect sizes and study precision. The central line represents the regression line, and the shaded region represents the 95% confidence interval. ESBL = Extended-spectrum β-lactamase; REML = Restricted maximum likelihood.

Substantial heterogeneity was observed, with τ² = 0.46, I² = 95.87%, and H² = 24.20. Cochran's Q test indicated statistically significant heterogeneity (Q(6) = 119.96, p < 0.001). The overall effect was also statistically significant (z = 2.93, p < 0.01).

The Galbraith plot (Figure 4B) showed broad dispersion of study estimates around the regression line, further demonstrating substantial between-study variability. However, all study estimates remained within the 95% confidence bounds, indicating no extreme outliers.

Sensitivity analysis restricted to phenotypically confirmed ESBL-producing *Vibrio* isolates included three studies comprising four species-level datasets and 212 isolates tested using the combined-disk method or double-disk synergy test. The pooled prevalence decreased to 12.5% (95% CI: 3.9–33.3%) compared with the overall estimate of 19.4% (95% CI: 3.2–43.7%).

### Pooled phenotypic resistance, MDR, resistance determinants, and virulence-associated genes

AST covered multiple antimicrobial classes, including aminoglycosides, β-lactams with or without β-lactamase inhibitors, carbapenems, macrolides, quinolones, tetracyclines, polymyxins, sulfonamides, phenicols, and nitrofurans (Table S4).

Among β-lactams, pooled resistance estimates were highest for ampicillin/sulbactam (74.5%, 95% CI: 37.5–98.3%), ampicillin (73.6%, 95% CI: 58.4–86.4%), and amoxicillin (60.9%, 95% CI: 12.0–98.8%). Resistance to amoxicillin/clavulanic acid was substantially lower at 8.3% (95% CI: 2.2–17.0%). Among cephalosporins, cefuroxime showed the highest pooled resistance estimate at 51.1% (95% CI: 15.8–85.8%), whereas overall pooled resistance to cefotaxime, cefepime, ceftazidime, and ceftriaxone remained below 20%.

Among aminoglycosides, pooled resistance was highest for kanamycin (47.8%, 95% CI: 24.2–71.8%) and streptomycin (32.6%, 95% CI: 10.0–60.6%), whereas lower estimates were observed for amikacin (8.6%, 95% CI: 1.6–19.3%) and gentamicin (1.6%, 95% CI: 0–5.2%). For macrolides, pooled resistance was 28.5% (95% CI: 3.9–63.0%) for azithromycin and 23.0% (95% CI: 6.1–45.9%) for erythromycin.

Overall resistance to quinolones was comparatively low, including ciprofloxacin (5.3%, 95% CI: 2.0–9.9%), norfloxacin (2.8%, 95% CI: 0–9.1%), and ofloxacin (13.5%, 95% CI: 3.4–27.9%). No levofloxacin resistance was detected in the available datasets. Among tetracyclines, pooled resistance was 8.0% (95% CI: 2.7–15.3%) for tetracycline and 4.1% (95% CI: 0–13.1%) for oxytetracycline, while no minocycline resistance was detected. Nitrofurantoin resistance was estimated at 79.6% (95% CI: 34.2–100.0%) based on African studies only. Among polymyxins, pooled resistance was 66.4% (95% CI: 7.7–100.0%) for polymyxin B and 57.7% (95% CI: 15.2–94.4%) for colistin.

The overall pooled MDR prevalence was 31.5% (95% CI: 6.1–64.7%). Continent-specific estimates were 100.0% (95% CI: 95.6–100.0%) in Africa and 23.3% (95% CI: 3.0–53.7%) in Asia, although the number of contributing studies differed among strata.

Among β-lactamase resistance determinants, *bla*_TEM _was the most frequently detected gene, with a pooled prevalence of 33.8% (95% CI: 7.0–67.4%). Carbapenemase-associated genes were also detected, including *bla*_VIM_ (15.5%, 95% CI: 0.7–41.7%) and *bla*_IMP _(9.1%, 95% CI: 4.3–15.2%). Pooled prevalence estimates were lower for *bla*OXA-48 (4.4%, 95% CI: 1.3–8.9%), *bla*_KPC_ (1.7%, 95% CI: 0–10.4%), and *bla*_SHV_ (1.6%, 95% CI: 0–9.2%). Among non-β-lactam resistance determinants, *aadA* was detected in 18.7% of isolates (95% CI: 2.7–43.1%), whereas *strA* and *sul2* were each detected in 4.9% (95% CI: 0.6–12.0%). The pooled prevalence of *tetA* was 6.8% (95% CI: 2.2–13.1%).

Virulence-associated genes were also identified. Their pooled prevalence estimates were 25.8% (95% CI: 0–99.9%) for *ctxA*, 26.3% (95% CI: 0–99.9%) for *tcpA*, 11.5% (95% CI: 0–57.6%) for *tdh*, and 0.3% (95% CI: 0–2.1%) for *trh*. Substantial between-study heterogeneity was observed for many phenotypic and genotypic outcomes (Table S4).

### Species-stratified phenotypic and genotypic AMR

Resistance patterns differed markedly among *Vibrio* species (Table S5). Ampicillin showed an overall pooled resistance prevalence of 73.6% (95% CI: 58.4–86.4%). Species-specific resistance was particularly high in *V. alginolyticus* (97.8%, n = 68), *V. mimicus* (95.0%, n = 40), *V. fluvialis* (87.8%, n = 41), and *V. parahaemolyticus* (84.1%, n = 974) (Figure 5), whereas the pooled estimate for *V. cholerae* was lower at 24.1% (n = 705).

Resistance to amoxicillin/clavulanic acid remained low overall (8.3%, 95% CI: 2.2–17.0%). Species-specific estimates were higher in *V. navarrensis* (17.4%, n = 46) and *V. parahaemolyticus* (16.3%, n = 203; 9.4%, n = 64), whereas no resistance was reported in *V. alginolyticus* (n = 21), *V. cholerae* (n = 20), or *V. vulnificus* (n = 31).

Among third- and fourth-generation cephalosporins, overall pooled resistance to cefotaxime was 16.2% (95% CI: 5.9–29.9%; n = 2,273). Higher species-specific estimates were observed for unspecified *Vibrio* spp. (36.6%, n = 643) and *V. scophthalmi* (57.6%, n = 33). Overall ceftazidime resistance was 7.2% (95% CI: 1.7–15.6%), although resistance reached 82.1% in *V. cholerae* O1 (n = 39). Ceftriaxone resistance was 13.2% overall (95% CI: 0.6–35.5%), with higher estimates in unspecified *Vibrio* spp. (62.3%, n = 59) and *V. scophthalmi* (33.3%, n = 33), whereas resistance in *V. parahaemolyticus* was 0.4% (n = 214).

Macrolide resistance also varied among species. Azithromycin resistance was 28.5% overall (95% CI: 3.9–63.0%) and reached 100.0% in *V. fluvialis*, *V. mimicus*, and *V. vulnificus*. Pooled erythromycin resistance was 23.0% (95% CI: 6.1–45.9%), with a higher estimate in *V. alginolyticus* (76.9%).

For quinolones, pooled ciprofloxacin resistance was 5.3% (95% CI: 2.0–9.9%), with higher estimates in *V. alginolyticus* (34.1%) and *V. fluvialis* (26.8%) than in *V. cholerae* (0.8%) and unspecified *Vibrio* spp. (2.0%). Nalidixic acid resistance was 31.7% (95% CI: 7.4–62.8%) overall and ranged from 95.2%–100.0% in *V. cholerae* O1 to 0.4% in *V. parahaemolyticus*. No levofloxacin resistance was detected (0%, 95% CI: 0–0.4%).

Among polymyxins, colistin resistance was 57.7% (95% CI: 15.2–94.4%), including 93.5% in *V. vulnificus* and 74.1% in *V. diabolicus*, compared with 7.0% in unspecified *Vibrio* spp. Polymyxin B resistance was 66.4% (95% CI: 7.7–100.0%) overall and reached 100.0% in *V. fluvialis*, *V. mimicus*, and *V. vulnificus*, whereas no resistance was detected in *V. cholerae*.

The overall pooled prevalence of MDR was 31.5% (95% CI: 6.1–64.7%). No MDR was detected in *V. alginolyticus* (0%, n = 21) or *V. navarrensis* (0%, n = 23), whereas MDR prevalence reached 100.0% in two *V. cholerae* O1 datasets (n = 39 and n = 22) and ranged from 18.3% to 28.1% in *V. parahaemolyticus*.

The prevalence of resistance-associated genes also varied among species. *bla*_TEM_ had an overall pooled prevalence of 33.8% (95% CI: 7.0–67.4%), with high estimates in *V. fluvialis* (85.4%) and *V. mimicus* (95.0%), whereas one *V. parahaemolyticus* stratum reported no detection. *bla*OXA-48 remained infrequent overall (4.4%, 95% CI: 1.3–8.9%), while *bla*_PER_ was not detected. Among other resistance determinants, *strA* and *sul2* each had an overall pooled prevalence of 4.9%, and *tetA* was detected in 6.8% of isolates (95% CI: 2.2–13.1%).

**Figure 5:**
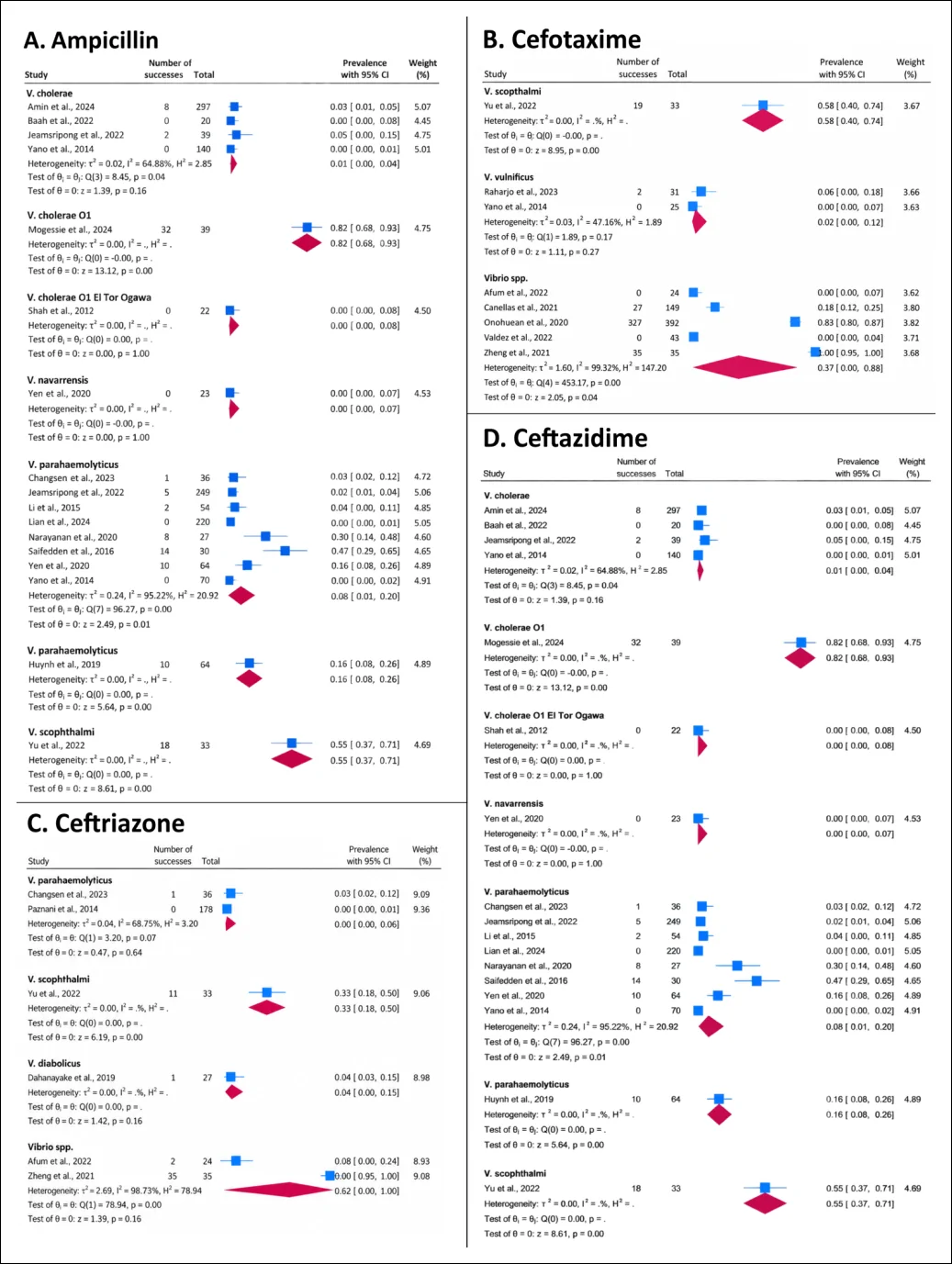
Species-specific resistance of *Vibrio* spp. to ampicillin and third-generation cephalosporins.

## DISCUSSION

### Pooled prevalence of *Vibrio* spp. and heterogeneity

This systematic review and meta-analysis provides a quantitative One Health synthesis of *Vibrio* spp. occurrence across human, animal, and environmental sources, extending previous evidence that was more restricted in scope and highlighting substantial geographic and species-level heterogeneity. The detection of *Vibrio* spp. across all three One Health sectors emphasizes their importance at the interface of aquatic ecosystems, food production, animal health, and human health. Most samples originated from animal sources, particularly aquatic animals, consistent with the close ecological association of *Vibrio* spp. with aquatic environments and the potential role of seafood and other animal-derived products in human exposure [58, 59]. The inclusion of human isolates further demonstrates the clinical relevance of antimicrobial-resistant *Vibrio* spp. [59, 60]. In contrast, the comparatively limited representation of environmental samples identifies an important surveillance gap and supports more balanced monitoring across human, animal, and environmental sectors.

The pooled prevalence of *Vibrio* spp. was 48.3%, although substantial between-study heterogeneity was observed (I² = 99.37%, p < 0.001). This heterogeneity likely reflects differences in geographic region, sample source, aquatic production systems, target *Vibrio* species, sampling periods, environmental conditions, and laboratory detection methods among the included studies. Prevalence differed significantly by continent, with a higher pooled estimate in Asia than in Africa. The overall pooled estimate was slightly higher than previously reported species-specific prevalence estimates, including 46.0% for *V. parahaemolyticus* in shrimp and 10.5% for *V. vulnificus* in seafood in Asia [3, 61]. These differences may partly reflect the broader scope of the present analysis, which combined multiple *Vibrio* species and heterogeneous sample sources rather than focusing on a single species or commodity.

Accordingly, the pooled prevalence should be interpreted as a descriptive summary of the available evidence rather than as a universally representative estimate of *Vibrio* occurrence. Greater emphasis should be placed on study-specific and subgroup estimates because of the substantial heterogeneity and geographic imbalance in the evidence base. The leave-one-out sensitivity analysis showed that no single influential study drove the pooled prevalence. Although funnel plot asymmetry suggested possible publication bias or small-study effects, persistent heterogeneity after sensitivity analysis indicates that the asymmetry may also reflect genuine differences among study populations and methodologies. Publication bias therefore cannot be excluded, but its interpretation should remain cautious. Future studies using standardized sampling frameworks, species identification procedures, and microbiological methods would improve comparability among geographic regions and strengthen pooled prevalence estimates.

### Pooled prevalence of ESBL-producing *Vibrio* spp.

The pooled prevalence of ESBL-producing Vibrio spp. was 19.4% (95% CI: 3.2–43.7%), indicating that ESBL-associated resistance occurs in a notable proportion of the isolates represented in the available literature. This finding is important because *Vibrio* spp. have historically been regarded as comparatively susceptible to several extended-spectrum cephalosporins, whereas increasing detection of acquired β-lactam resistance determinants may compromise this susceptibility [7, 20, 21]. Nevertheless, the estimate should not be interpreted as evidence that approximately one-fifth of all *Vibrio* isolates globally are ESBL producers because the evidence base was limited and highly heterogeneous.

Substantial heterogeneity was observed for ESBL prevalence (I² = 95.87%), indicating pronounced variation among studies. The pooled estimate therefore represents a context-dependent summary rather than a stable global prevalence. Differences in geographic setting, sample source, antimicrobial use practices, environmental contamination, study population, AST methodology, and criteria for defining ESBL production may contribute to this variation [14, 21, 62]. The concentration of available studies in Asia and parts of Africa further limits global representativeness.

The sensitivity analysis provided additional evidence of the influence of ESBL classification methods. When the meta-analysis was restricted to phenotypically confirmed ESBL-producing *Vibrio* isolates, pooled prevalence decreased from 19.4% to 12.5%. This finding suggests that inclusion of molecularly detected ESBL-associated genes without corresponding phenotypic confirmation can influence pooled prevalence estimates. Detection of an ESBL-associated gene does not necessarily establish phenotypic expression under the testing conditions used. Consequently, consider differences in ESBL definitions, confirmatory procedures, and reporting practices when comparing prevalence estimates across studies.

### Resistance determinants and virulence-associated genes

The geographic concentration of studies in Asia and Africa may reflect, at least in part, the importance of aquaculture intensity, urbanization, wastewater discharge, and other anthropogenic pressures in shaping aquatic AMR epidemiology [16, 18, 63]. Aquatic environments exposed to antimicrobial residues and resistant bacteria can provide ecological opportunities for persistence and horizontal transfer of resistance determinants. Mobile genetic elements, including plasmids, integrons, and SXT/R391-related elements, have been implicated in the dissemination of AMR among aquatic bacteria [18, 22, 64].

Among the resistance determinants evaluated, *bla*_TEM_ showed the highest pooled prevalence among the reported β-lactamase genes at 33.8%. Other clinically important determinants, including *bla*_VIM_, *bla*_IMP_, *bla*_OXA-48_, *bla*_KPC_, and *bla*_SHV_, were also identified, although generally at lower pooled prevalences. These findings support the potential role of aquatic *Vibrio* populations as reservoirs of clinically relevant resistance determinants. The detection of resistant *Vibrio* spp. in aquatic animals, seafood, environmental samples, and human clinical sources further demonstrates the interconnected nature of resistance across the One Health interface [15, 23, 58].

The present meta-analysis also identified virulence-associated genes, including *ctxA*, *tcpA*, *tdh*, and *trh*. Their occurrence alongside resistance determinants in the overall evidence base is of concern because pathogenicity and AMR can coexist within *Vibrio* populations. However, the present pooled analyses do not establish that resistance and virulence genes were necessarily carried by the same isolates unless the original studies explicitly demonstrated this. Therefore, the evidence should not be interpreted as proving the widespread emergence of simultaneously hypervirulent and multidrug-resistant lineages.

The ecological relationships among antimicrobial exposure, mobile genetic elements, aquatic microbial communities, food production, and human exposure are summarized conceptually in Figure 6.

**Figure 6:**
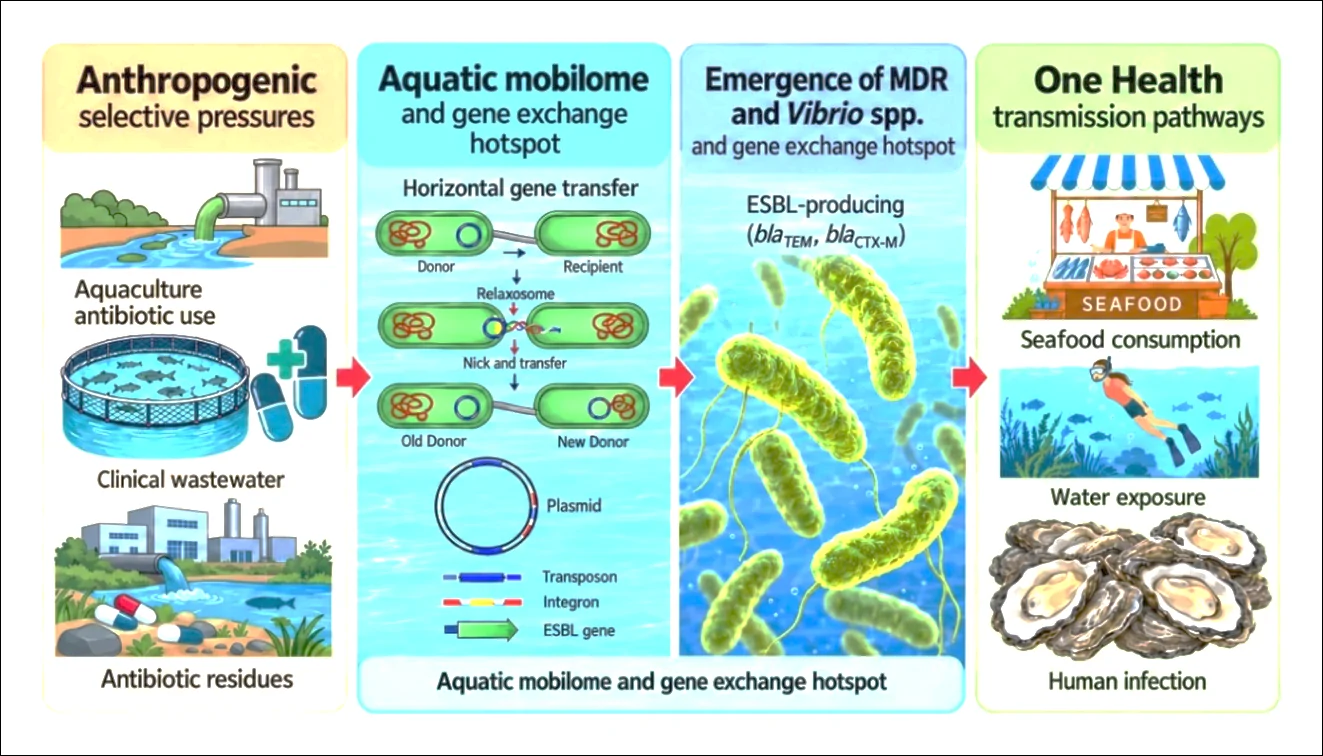
Conceptual model of the emergence, amplification, and transmission of AMR in *Vibrio* spp. across the One Health interface. Anthropogenic activities, including antimicrobial use in aquaculture, wastewater discharge, and agricultural runoff, can introduce antimicrobial residues and resistant bacteria into aquatic environments. These conditions may facilitate persistence and horizontal transfer of resistance determinants through mobile genetic elements, including plasmids, integrons, and SXT/R391-related elements. Antimicrobial-resistant *Vibrio* spp. may subsequently circulate among aquatic environments, aquaculture systems, seafood, animals, and humans through foodborne and environmental exposure pathways. This graphical illustration was generated with the assistance of ChatGPT (OpenAI, San Francisco, CA, USA) and the final figure was reviewed and verified by the authors for scientific accuracy. AMR = Antimicrobial resistance; MDR = Multidrug resistance; SXT/R391 = SXT/R391 family of integrative and conjugative elements.

### Phenotypic AMR

The phenotypic resistance profile varied markedly across antimicrobial classes. Ampicillin resistance was particularly high, with a pooled estimate of 73.6%. This finding is consistent with the frequent occurrence of β-lactam resistance among *Vibrio* spp. and may reflect both intrinsic and acquired resistance mechanisms [7, 8]. The pooled prevalence of ESBL-producing isolates and detection of ESBL-associated genes indicate that acquired β-lactam resistance mechanisms also contribute to the resistance landscape.

Resistance to cefuroxime was also relatively high at 51.1%, whereas overall resistance to cefotaxime, cefepime, ceftazidime, and ceftriaxone remained below 20%. These differences among β-lactam agents show that resistance cannot be generalized across an entire antimicrobial class and should be interpreted by individual antimicrobial agent and *Vibrio* species.

In contrast, overall ciprofloxacin resistance was comparatively low at 5.3%. Resistance to other quinolones was also generally lower than resistance to several β-lactams. However, the included studies did not directly evaluate the mechanisms behind these differences as part of the meta-analysis. Therefore, the observed disparity should not be attributed solely to differences between chromosomal mutation and horizontal gene transfer without supporting molecular evidence.

The pooled MDR prevalence was 31.5%, although substantial regional variation was observed. The African estimate was particularly high; however, this result should be interpreted cautiously because of the limited number of contributing studies and the wide differences in sampling and study characteristics. Regional differences in antimicrobial use, wastewater management, aquaculture practices, environmental contamination, and surveillance intensity may contribute to variation in MDR occurrence [8, 21].

The detection of carbapenemase-associated genes such as *bla*_VIM_ and *bla*_IMP_ is particularly noteworthy because these determinants are clinically important and may circulate through mobile genetic elements [17, 24]. Their identification in aquatic *Vibrio* spp. supports continued monitoring of aquatic ecosystems and seafood production systems as potential reservoirs and transmission pathways for clinically important AMR determinants [15, 23]. Nevertheless, the present findings demonstrate occurrence rather than direct transmission of these genes between environmental, animal, and human populations.

### Species-specific AMR

Marked interspecies variation was observed across multiple antimicrobial classes, emphasizing the limitations of genus-level generalization. Ampicillin resistance was particularly high in *V. alginolyticus*, *V. mimicus*, *V. fluvialis*, and *V. parahaemolyticus*, whereas the pooled estimate for *V. cholerae* was substantially lower. Similarly, ceftazidime resistance reached 82.1% in *V. cholerae* O1 in one dataset, whereas resistance remained much lower in *V. parahaemolyticus*. Cefotaxime and ceftriaxone resistance were also comparatively high in *V. scophthalmi* relative to several other species.

Species-specific differences were also evident for macrolides, quinolones, and polymyxins. For example, azithromycin resistance reached 100% in some datasets for *V. fluvialis*, *V. mimicus*, and *V. vulnificus*, whereas pooled ciprofloxacin resistance remained low overall but was substantially higher in *V. alginolyticus* and *V. fluvialis*. These patterns indicate that AMR within the genus *Vibrio* is highly species dependent and may additionally vary according to geographic setting, source population, and antimicrobial exposure.

These findings support species-level AMR surveillance rather than reliance solely on genus-level summaries. From veterinary and public health perspectives, improved species identification, standardized AST, and molecular characterization would provide more informative data for antimicrobial stewardship, aquaculture biosecurity, food safety surveillance, and clinical risk assessment [11, 15, 17].

### Contribution of the present study and One Health implications

This systematic review and meta-analysis integrates epidemiological, phenotypic, and genotypic evidence on ESBL-producing and antimicrobial-resistant *Vibrio* spp. across human, animal, food, and environmental sources. The pooled ESBL prevalence of 19.4%, high resistance to several β-lactam agents, overall MDR prevalence of 31.5%, and detection of clinically important resistance determinants demonstrate that AMR in *Vibrio* spp. warrants continued attention within a One Health framework.

The findings have practical implications for both clinical and veterinary settings. Empirical antimicrobial treatment of vibriosis should account for local susceptibility patterns, and AST-guided therapy should be used where feasible. In aquaculture, antimicrobial stewardship should emphasize appropriate antimicrobial selection, avoiding unnecessary exposure, biosecurity, disease prevention, and resistance surveillance within production systems.

A major contribution of the present study is the demonstration that resistance patterns are highly heterogeneous across geographic regions and *Vibrio* species. This heterogeneity indicates that a single global pooled estimate cannot adequately describe the complexity of AMR across aquatic systems. Surveillance programs should therefore integrate species-level identification, standardized phenotypic testing, molecular detection of resistance determinants, and source-specific epidemiological information.

The One Health perspective matters because aquatic environments connect antimicrobial use, wastewater contamination, food production, animal populations, and human exposure. The available evidence supports strengthening coordinated surveillance across these interfaces rather than monitoring clinical isolates in isolation. Future surveillance incorporating genomic approaches could further clarify the relatedness of resistant isolates, mobile genetic elements, and potential transmission pathways among environmental, animal, food, and human reservoirs.

### Strengths of this study

This study provides a comprehensive synthesis of the available evidence on ESBL-producing *Vibrio* spp. across environmental, seafood, aquaculture, animal, and clinical sources. A major strength is integrating phenotypic AMR profiles with genotypic data on ESBL-associated and other resistance determinants, enabling a broader assessment of resistance characteristics across diverse *Vibrio* species and source populations. The study also evaluates species-specific AMR patterns and geographic variation, which are important for identifying areas and taxa in which resistance may be more frequently reported.

Another strength is the application of a One Health framework to integrate evidence across aquatic environments, food production systems, animals, and humans. This approach provides a more comprehensive perspective on the epidemiology of antimicrobial-resistant *Vibrio* spp. than analyses restricted to a single host or environmental compartment. In addition, subgroup analyses, sensitivity analyses, risk-of-bias assessment, and separate evaluation of phenotypic and genotypic outcomes strengthen interpretation of the pooled estimates and highlight how study design, geographic setting, species distribution, and ESBL-definition criteria may influence the findings.

### Limitations of the study

This study has several limitations. First, substantial heterogeneity was observed across many pooled outcomes. This heterogeneity may reflect differences in geographic location, sample source, target *Vibrio* species, sampling strategy, laboratory detection procedures, AST methods, and interpretative criteria. In particular, antimicrobial susceptibility classification may have varied across studies because different editions of CLSI and EUCAST guidelines, as well as human- or veterinary-oriented interpretative standards, were used. Such methodological variability limits direct comparability among studies and may contribute to uncertainty in pooled estimates.

Second, only English-language publications were included, which may have introduced language bias and excluded relevant evidence published in other languages. Third, although multiple information sources were searched, the inclusion of Google Scholar may have introduced additional variability because its indexing, ranking, and reproducibility differ from those of conventional bibliographic databases.

Fourth, geographic representation was uneven. Most available evidence originated from Asia and Africa, whereas Europe, North America, South America, and other regions were comparatively underrepresented. Consequently, regional pooled estimates should not be interpreted as equally representative across continents, and the overall findings should not be generalized uncritically to regions with limited data.

Fifth, selective reporting of resistant isolates, specific antimicrobial agents, ESBL-associated genes, or statistically notable findings may have influenced the available evidence base and could have contributed to overestimation of some pooled resistance or prevalence estimates. Formal assessment of publication bias was also constrained by the small number of contributing studies for several outcomes, reducing the reliability of funnel plot interpretation and the statistical power of Egger's regression test. Potential selective reporting therefore had to be considered qualitatively when interpreting the findings.

Finally, the included studies differed in their definitions and confirmation methods for ESBL production. Some studies relied on phenotypic confirmation, whereas others reported molecular detection of ESBL-associated genes. Because the presence of a resistance-associated gene does not necessarily indicate phenotypic expression, combining these approaches may have influenced pooled prevalence estimates. This concern was partly addressed through sensitivity analysis restricted to phenotypically confirmed ESBL-producing isolates, but methodological heterogeneity remains an important limitation. Overall, the findings should therefore be interpreted cautiously, particularly when making subgroup comparisons or extrapolating the pooled estimates to a global population.

## CONCLUSION

This systematic review and meta-analysis demonstrates that antimicrobial-resistant *Vibrio* spp., including ESBL-producing isolates, represent an important One Health concern across human, animal, food, and aquatic environmental interfaces. The pooled prevalence of *Vibrio* spp. was 48.3% (95% CI: 34.0–62.7%), but substantial heterogeneity (I² = 99.37%) and uneven geographic representation mean this estimate reflects the available evidence rather than a globally representative prevalence. ESBL-producing *Vibrio* spp. had a pooled prevalence of 19.4% (95% CI: 3.2–43.7%), which decreased to 12.5% (95% CI: 3.9–33.3%) when the analysis was restricted to phenotypically confirmed ESBL-producing isolates, highlighting the influence of ESBL definitions and detection methods on prevalence estimates. The pooled prevalence of MDR was 31.5%, further demonstrating the importance of AMR among the investigated *Vibrio* populations.

Phenotypic resistance varied considerably among antimicrobial classes and *Vibrio* species. Pooled resistance was particularly high for ampicillin (73.6%), ampicillin/sulbactam (74.5%), amoxicillin (60.9%), and cefuroxime (51.1%), whereas ciprofloxacin resistance remained comparatively low at 5.3%. Genotypic evidence also showed clinically important resistance determinants, with *bla*_TEM_ as the most commonly reported β-lactamase gene, along with *bla*_VIM_, *bla*_IMP_, *bla*_OXA-48_, *bla*_KPC_, and *bla*_SHV_. The marked geographic and species-specific variation observed across the analyses indicates that genus-level pooled estimates alone cannot adequately characterize the epidemiology of AMR in *Vibrio* spp.

Future research should prioritize geographically balanced, prospective surveillance integrating human, animal, seafood, aquaculture, and environmental sampling within coordinated One Health frameworks. Standardized sampling procedures, species identification methods, AST protocols, interpretative criteria, and phenotypic confirmation of ESBL production are needed to improve comparability among studies. Greater incorporation of whole-genome sequencing and other genomic approaches could characterize resistance determinants, virulence-associated genes, mobile genetic elements, and genetic relatedness among isolates and help determine whether resistant strains or resistance determinants are shared across One Health sectors. Longitudinal investigations linking antimicrobial use, wastewater contamination, aquaculture practices, environmental conditions, and AMR occurrence are also needed to clarify temporal trends and potential transmission pathways.

Overall, the findings demonstrate that ESBL production, MDR, and clinically important resistance determinants are present among *Vibrio* spp. across interconnected aquatic, food, animal, and human systems. The considerable heterogeneity and limited evidence from several geographic regions preclude unrestricted global generalization; nevertheless, the available evidence supports strengthening species-specific and source-specific AMR surveillance. Coordinated One Health monitoring, standardized susceptibility testing, antimicrobial stewardship in clinical and aquaculture settings, and genomic surveillance will be important for defining emerging resistance patterns and informing evidence-based strategies to reduce the potential dissemination and public health impact of antimicrobial-resistant *Vibrio* spp.

## DATA AVAILABILITY

The supplementary data can be made available from the corresponding author upon request.

## GENERATIVE AI DECLARATION

The authors declare that generative artificial intelligence (AI) tools were used solely to improve language, grammar, and readability during manuscript preparation.

## AUTHORS’ CONTRIBUTIONS

VT: Conceptualization, methodology, investigation, data collection, formal analysis, data interpretation, project administration, visualization, writing – original draft, and writing – review and editing. SA: Methodology, investigation, data collection, and writing – review and editing. NR: Methodology and writing – review and editing. MTK: Investigation and writing – original draft. SJ: Conceptualization, methodology, investigation, data collection, formal analysis, data interpretation, supervision, and writing – review and editing. All authors have read and approved the final version of the manuscript.

## References

[1] Murray CJ, Ikuta KS, Sharara F, Swetschinski L, Aguilar GR, Gray A (2022). Global burden of bacterial antimicrobial resistance in 2019: a systematic analysis. Lancet.

[2] Salam MA, Al-Amin MY, Salam MT, Pawar JS, Akhter N, Rabaan AA (2023). Antimicrobial resistance: a growing serious threat for global public health. Healthcare.

[3] Thaotumpitak V, Odoi JO, Anuntawirun S, Jeamsripong S (2024). Meta-analysis and systematic review of phenotypic and genotypic antimicrobial resistance and virulence factors in Vibrio parahaemolyticus isolated from shrimp. Antibiotics.

[4] Ilic I, Ilic M (2023). Global patterns of trends in cholera mortality. Trop Med Infect Dis.

[5] Castanheira M, Simner PJ, Bradford PA (2021). Extended-spectrum β-lactamases: an update on their characteristics, epidemiology and detection. JAC Antimicrob Resist.

[6] Qibtiyah M, Safiro A, Nuzulistina E, Addimaysqi R, Wahyuhadi J, Khotib J (2025). Mortality outcomes of extended-spectrum β-lactamase in bacteremia with carbapenems and other antibiotics: a systematic review. J Adv Pharm Technol Res.

[7] Dutta D, Kaushik A, Kumar D, Bag S (2021). Foodborne pathogenic vibrios: antimicrobial resistance. Front Microbiol.

[8] Gxalo O, Digban TO, Igere BE, Olapade OA, Okoh AI, Nwodo UU (2021). Virulence and antibiotic resistance characteristics of Vibrio isolates from rustic environmental freshwaters. Front Cell Infect Microbiol.

[9] Kamble MT, Soowannayan C, Chaicherd S, Medhe SV, Rudtanatip T, Pissuwan D (2024). Bimetallic nanoparticles with sulfated galactan eliminate Vibrio parahaemolyticus in shrimp Penaeus vannamei. Fish Shellfish Immunol.

[10] Kamble MT, Rudtanatip T, Soowannayan C, Nambunruang B, Medhe SV, Wongprasert K (2022). Depolymerized fractions of sulfated galactans extracted from Gracilaria fisheri and their antibacterial activity against Vibrio parahaemolyticus and Vibrio harveyi. Mar Drugs.

[11] EFSA Panel on Biological Hazards (BIOHAZ), Koutsoumanis K, Allende A, Alvarez-Ordóñez A, Bolton D, Bover-Cid S (2024). Public health aspects of Vibrio spp. related to the consumption of seafood in the EU. EFSA J.

[12] Cai R, Zhen M, Guan Z, Li M, Liao Q, Liu X (2021). New atypical manifestations and prognostic factors of Vibrio vulnificus infection: a 10-year retrospective study. Jpn J Infect Dis.

[13] Loo KY, Law JWF, Tan LTH, Pusparajah P, Wong SH, Chan KG (2023). The burden of Vibrio sp. infections—a scoping review. Prog Microbes Mol Biol.

[14] Ekundayo TC, Tabit FT (2025). Occurrence and antibiotic resistance risk burden of Vibrio mimicus isolates from seafood and aquatic environments. Antibiotics.

[15] Hirshfeld B, Lavelle K, Lee KY, Atwill ER, Kiang D, Bolkenov B (2023). Prevalence and antimicrobial resistance profiles of Vibrio spp. and Enterococcus spp. in retail shrimp in Northern California. Front Microbiol.

[16] Sony M, Sumithra T, Anusree V, Amala P, Reshma K, Alex S (2021). Antimicrobial resistance and virulence characteristics of Vibrio vulnificus, Vibrio parahaemolyticus and Vibrio harveyi from natural disease outbreaks of marine/estuarine fishes. Aquaculture.

[17] Sanches-Fernandes GMM, Sá-Correia I, Costa R (2022). Vibriosis outbreaks in aquaculture: addressing environmental and public health concerns and preventive therapies using gilthead seabream farming as a model system. Front Microbiol.

[18] Zheng Z, Ye L, Xu Y, Chan EWC, Chen S (2024). Dynamics of antimicrobial resistance and genomic characteristics of foodborne Vibrio spp. in Southern China (2013–2022). J Hazard Mater.

[19] Chen H, Fang Q, Zheng L, Wu Y, Liang W (2025). Clonal dynamics, virulence genes, antimicrobial resistance, and early diagnostic indicators of bloodstream infections of Vibrio vulnificus in a hospital in Eastern China from 2021 to 2024. Infect Drug Resist.

[20] Sivan G, Hridya VK, Sukumaran DP, Abdulla MH (2024). Exploring extended-spectrum beta lactamase resistance in Vibrio parahaemolyticus and Vibrio cholerae within the tropical mangrove ecosystem of southwest India. Braz J Microbiol.

[21] Onohuean H, Agwu E, Nwodo UU (2022). Systematic review and meta-analysis of environmental Vibrio species–antibiotic resistance. Heliyon.

[22] Bhat BA, Mir RA, Qadri H, Dhiman R, Almilaibary A, Alkhanani M (2023). Integrons in the development of antimicrobial resistance: critical review and perspectives. Front Microbiol.

[23] Valero A, Arroyo-López FN, Cabo ML, Chen S, Pérez-Díaz IM (2021). Editorial: Vibrio species in the food processing chain. Front Microbiol.

[24] Rafei R, Osman M, Kassem II, Dabboussi F, Weill FX, Hamze M (2024). Spotlight on the epidemiology and antimicrobial susceptibility profiles of Vibrio species in the MENA region, 2000–2023. Future Microbiol.

[25] Page MJ, McKenzie JE, Bossuyt PM, Boutron I, Hoffmann TC, Mulrow CD (2021). The PRISMA 2020 statement: an updated guideline for reporting systematic reviews. BMJ.

[26] Morgan RL, Whaley P, Thayer KA, Schünemann HJ (2018). Identifying the PECO: a framework for formulating good questions to explore the association of environmental and other exposures with health outcomes. Environ Int.

[27] Munn Z, Moola S, Lisy K, Riitano D, Tufanaru C (2015). Methodological guidance for systematic reviews of observational epidemiological studies reporting prevalence and cumulative incidence data. Int J Evid Based Healthc.

[28] Afum T, Asandem DA, Asare P, Asante-Poku A, Mensah GI, Musah AB (2022). Diarrhea-causing bacteria and their antibiotic resistance patterns among diarrhea patients from Ghana. Front Microbiol.

[29] Amin MB, Talukdar PK, Sraboni AS, Islam MR, Mahmud ZH, Berendes D (2024). Prevalence and antimicrobial resistance of major foodborne pathogens isolated from pangas and tilapia fish sold in retail markets of Dhaka city, Bangladesh. Int J Food Microbiol.

[30] Baah DA, Kotey FCN, Dayie NTKD, Codjoe FS, Tetteh-Quarcoo PB, Donkor ES (2022). Multidrug-resistant Gram-negative bacteria contaminating raw meat sold in Accra, Ghana. Pathogens.

[31] Baron S, Larvor E, Chevalier S, Jouy E, Kempf I, Granier SA (2017). Antimicrobial susceptibility among urban wastewater and wild shellfish isolates of non-O1/non-O139 Vibrio cholerae from La Rance Estuary (Brittany, France). Front Microbiol.

[32] Beshiru A, Okareh OT, Okoh AI, Igbinosa EO (2020). Detection of antibiotic resistance and virulence genes of Vibrio strains isolated from ready-to-eat shrimps in Delta and Edo States, Nigeria. J Appl Microbiol.

[33] Canellas AL, Lopes IR, Mello MP, Paranhos R, de Oliveira BFR, Laport MS (2021). Vibrio species in an urban tropical estuary: antimicrobial susceptibility, interaction with environmental parameters, and possible public health outcomes. Microorganisms.

[34] Changsen C, Likhitrattanapisal S, Lunha K, Chumpol W, Jiemsup S, Prachumwat A (2023). Incidence, genetic diversity, and antimicrobial resistance profiles of Vibrio parahaemolyticus in seafood in Bangkok and eastern Thailand. PeerJ.

[35] Chiyangi H, Muma JB, Malama S, Manyahi J, Abade A, Kwenda G (2017). Identification and antimicrobial resistance patterns of bacterial enteropathogens from children aged 0–59 months at the University Teaching Hospital, Lusaka, Zambia: a prospective cross sectional study. BMC Infect Dis.

[36] Dahanayake P, Hossain S, Wickramanayake M, Wimalasena S, Heo GJ (2020). Manila clam (Ruditapes philippinarum) marketed in Korea as a source of vibrios harbouring virulence and β-lactam resistance genes. Lett Appl Microbiol.

[37] Gupta P, Modgil V, Kant V, Kaur H, Narayan C, Mahindroo J (2022). Phenotypic and genotypic characterization of antimicrobial resistance in clinical isolates of Vibrio cholerae over a decade (2002–2016). Indian J Med Microbiol.

[38] Huynh TNT, Vo TTA, Nguyen TPY, Nguyen VC (2019). Prevalence, antimicrobial resistance profiles and virulence genes of Vibrio spp. isolated from shrimp retails in Ho Chi Minh City (Vietnam). J Agric Dev.

[39] Jeamsripong S, Thaotumpitak V, Anuntawirun S, Roongrojmongkhon N, Atwill ER, Hinthong W (2022). Molecular epidemiology of antimicrobial resistance and virulence profiles of Escherichia coli, Salmonella spp., and Vibrio spp. isolated from coastal seawater for aquaculture. Antibiotics.

[40] Jubyda FT, Nahar KS, Barman I, Johura FT, Islam MT, Sultana M (2023). Vibrio cholerae O1 associated with recent endemic cholera shows temporal changes in serotype, genotype, and drug-resistance patterns in Bangladesh. Gut Pathog.

[41] Li R, Lin D, Chen K, Wong MH, Chen S (2015). First detection of AmpC β-lactamase blaCMY-2 on a conjugative IncA/C plasmid in a Vibrio parahaemolyticus isolate of food origin. Antimicrob Agents Chemother.

[42] Lian XQ, Liu GD, Huang MF, Fan QH, Lin ZD (2024). Antimicrobial resistance, virulence factors and phylogenetic profiles of Vibrio parahaemolyticus in the eastern coast of Shenzhen. Front Microbiol.

[43] Lajnef R, Morinigo MA, Hassen A, Abdallah LB (2024). Resistance profiling, evolution of biofilm formation and genetic diversity of Vibrio alginolyticus isolated from aquaculture systems. INSTM Bull Mar Freshw Sci.

[44] Mogessie H, Legesse M, Hailu AF, Teklehaymanot T, Alemayehu H, Abubeker R (2024). Vibrio cholerae O1 and Escherichia coli O157:H7 from drinking water and wastewater in Addis Ababa, Ethiopia. BMC Microbiol.

[45] Narayanan SV, Joseph TC, Peeralil S, Koombankallil R, Vaiyapuri M, Mothadaka MP (2020). Tropical shrimp aquaculture farms harbour pathogenic Vibrio parahaemolyticus with high genetic diversity and carbapenam resistance. Mar Pollut Bull.

[46] Onohuean H, Okoh AI, Nwodo UU (2022). Antibiogram signatures of Vibrio species recovered from surface waters in South Western districts of Uganda: implications for environmental pollution and infection control. Sci Total Environ.

[47] Pazhani GP, Bhowmik SK, Ghosh S, Guin S, Dutta S, Rajendran K (2014). Trends in the epidemiology of pandemic and non-pandemic strains of Vibrio parahaemolyticus isolated from diarrheal patients in Kolkata, India. PLoS Negl Trop Dis.

[48] Raharjo HM, Budiyansah H, Mursalim MF, Chokmangmeepisarn P, Sakulworakan R, Debnath PP (2023). The first evidence of blaCTX-M-55, QnrVC5, and novel insight into the genome of MDR Vibrio vulnificus isolated from Asian sea bass (Lates calcarifer) identified by resistome analysis. Aquaculture.

[49] Saifedden G, Farinazleen G, Nor-Khaizura A, Kayali A, Nakaguchi Y, Nishibuchi M (2016). Antibiotic susceptibility profile of Vibrio parahaemolyticus isolated from shrimp in Selangor, Malaysia. Int Food Res J.

[50] Shah BK, Sharma S, Shakya G, Upadhyay BP (2012). Multiple drug resistant Vibrio cholerae, Salmonella and Shigella from Nepalgunj cholera outbreak and different hospitals of Nepal. Nepalese J Biosci.

[51] Tate H, Ayers S, Nyirabahizi E, Li C, Borenstein S, Young S (2022). Prevalence of antimicrobial resistance in select bacteria from retail seafood—United States, 2019. Front Microbiol.

[52] Thaotumpitak V, Sripradite J, Atwill ER, Jeamsripong S (2023). Emergence of colistin resistance and characterization of antimicrobial resistance and virulence factors of Aeromonas hydrophila, Salmonella spp., and Vibrio cholerae isolated from hybrid red tilapia cage culture. PeerJ.

[53] Yen NTP, Nhung NT, Van NTB, Van Cuong N, Chau LTT, Trinh HN (2020). Antimicrobial residues, non-typhoidal Salmonella, Vibrio spp. and associated microbiological hazards in retail shrimps purchased in Ho Chi Minh city (Vietnam). Food Control.

[54] Yano Y, Hamano K, Satomi M, Tsutsui I, Ban M, Aue-Umneoy D (2014). Prevalence and antimicrobial susceptibility of Vibrio species related to food safety isolated from shrimp cultured at inland ponds in Thailand. Food Control.

[55] Yu Y, Liu X, Wang Y, Liao M, Tang M, Rong X (2022). Antimicrobial resistance and genotype characteristics of Vibrio scophthalmi isolated from diseased mariculture fish intestines with typical inter-annual variability. Front Mar Sci.

[56] Valdez C, Costa C, Simões M, De Carvalho CC, Baptista T, Campos MJ (2022). Detection of mcr-1 gene in undefined Vibrio species isolated from clams. Microorganisms.

[57] Zheng Z, Ye L, Li R, Chen S (2021). Whole-genome sequencing of strains of Vibrio spp. from China reveals different genetic contexts of blaCTX-M-14 among diverse lineages. J Antimicrob Chemother.

[58] Orlić K, Vardić Smrzlić I, Gavrilović A, Jug-Dujaković J, Perić L, Kolda A (2025). Preliminary assessment of antimicrobial resistance in Vibrio spp. from the Adriatic Sea: spatial patterns and One Health implications. Reg Stud Mar Sci.

[59] Yang C, Li Y, Jiang M, Wang L, Jiang Y, Hu L (2022). Outbreak dynamics of foodborne pathogen Vibrio parahaemolyticus over a seventeen year period implies hidden reservoirs. Nat Microbiol.

[60] Bastin A, Knabe L, Simpson M Climate change and cholera: a review exploring the association between storm severity and global human Vibrio spp. incidence. Georgetown Med Rev. 2024;8(1).

[61] Tanveer M, Ntakiyisumba E, Won G (2024). Prevalence and risk factors of seafood-borne Vibrio vulnificus in Asia: a systematic review with meta-analysis and meta-regression. Front Microbiol.

[62] Ramatla T, Mafokwane T, Lekota K, Monyama M, Khasapane G, Serage N (2023). “One Health” perspective on prevalence of co-existing extended-spectrum β-lactamase (ESBL)-producing Escherichia coli and Klebsiella pneumoniae: a comprehensive systematic review and meta-analysis. Ann Clin Microbiol Antimicrob.

[63] Mitsuwan W, Boripun R, Saengsawang P, Intongead S, Boonplu S, Chanpakdee R (2025). Multidrug resistance, biofilm-forming ability, and molecular characterization of Vibrio species isolated from foods in Thailand. Antibiotics.

[64] Nonaka L, Masuda M, Yano H (2022). Atypical integrative element with strand-biased circularization activity assists interspecies antimicrobial resistance gene transfer from Vibrio alfacsensis. PLoS One.

